# Controlled and tuneable drug release from electrospun fibers and a non-invasive approach for cytotoxicity testing

**DOI:** 10.1038/s41598-019-40079-7

**Published:** 2019-03-05

**Authors:** G. Piccirillo, D. A. Carvajal Berrio, A. Laurita, A. Pepe, B. Bochicchio, K. Schenke-Layland, S. Hinderer

**Affiliations:** 1 Department of Science, University of Basilicata, 85100 Potenza, Italy; 2 Department of Women’s Health, Research Institute for Women’s Health, Eberhard-Karls-University Tübingen, 72076 Tübingen, Germany; 3 Department of Biophysical Chemistry, Natural and Medical Sciences Institute (NMI) at the University of Tübingen, 72770 Reutlingen, Germany; 4 Department of Medicine/Cardiology, Cardiovascular Research Laboratories, David Geffen School of Medicine at UCLA, Los Angeles, CA USA

## Abstract

Electrospinning is an attractive method to generate drug releasing systems. In this work, we encapsulated the cell death-inducing drug Diclofenac (DCF) in an electrospun poly-L-lactide (PLA) scaffold. The scaffold offers a system for a sustained and controlled delivery of the cytotoxic DCF over time making it clinically favourable by achieving a prolonged therapeutic effect. We exposed human dermal fibroblasts (HDFs) to the drug-eluting scaffold and employed multiphoton microscopy and fluorescence lifetime imaging microscopy. These methods were suitable for non-invasive and marker-independent assessment of the cytotoxic effects. Released DCF induced changes in cell morphology and glycolytic activity. Furthermore, we showed that drug release can be influenced by adding dimethyl sulfoxide as a co-solvent for electrospinning. Interestingly, without affecting the drug diffusion mechanism, the resulting PLA scaffolds showed altered fibre morphology and enhanced initial DCF burst release. The here described model could represent an interesting way to control the diffusion of encapsulated bio-active molecules and test them using a marker-independent, non-invasive approach.

## Introduction

Diclofenac (2-2-(2,6-dichloroanilino)phenylacetic acid, DCF) is one of the most sold and used non-steroidal anti-inflammatory drugs prescribed to millions of people worldwide^1,2^ for the treatment of osteoarthritis, rheumatoid arthritis^3,4^, and muscle pain^5^, as well as other applications^6^. DCF exhibits anti-cancer effects^7–10^ and is effective in the treatment of actinic keratosis^11^. DCF is a potent non-selective cyclooxygenase inhibitor^2,12^; however, its full functional activity is thought to be related to a more complex mechanism of action, which has been investigated over the recent years^12,13^ as well as toxic side-effects related to DCF therapies^14–21^. Since liver toxicity represents the most reported complication related to prolonged or high-dosage use of DCF, *in vitro* studies have mainly focused on hepatocytes. Studies with cultured hepatocytes from various species demonstrated that high DCF concentrations are able to induce acute cell injury^22–29^. Recently, *in vitro* toxicity of DCF has been also demonstrated in other cell-lines^30,31^.

Although the mechanism of action of DCF is widely known, the mechanism of acute cellular toxicity has not been clearly determined. Moreover, the relevance of the previous studies has been questioned since they used very high concentrations which don’t mimic a clinical therapeutic situation. While DCF hepato-^26–29,32–35^ and nephro-toxicity^17,18,21,36^ has been widely investigated, not that much is known about its activity as an anti-cancer drug^6–10,37^. For example, the mode of action of DCF in combination with hyaluronic acid in the local treatment of cutaneous actinic keratosis is largely elusive, but its chemotherapeutic activity could be associated with drug induced apoptosis^38,39^.

In this work we aimed to describe DCF induced cell death in human dermal fibroblasts (HDFs) using a new effective, non-destructive *in vitro* model. Herein, HDFs were incubated together with a DCF-loaded electrospun poly-L-lactide (PLA) scaffold, which ensured to obtain a controlled drug release over 24 hours. The DCF exposed cells were imaged using multiphoton microscopy (MPM) and their metabolic activity was investigated using fluorescence lifetime imaging microscopy (FLIM). For the FLIM and MPM analyses reduced (phosphorylated) nicotinamide adenine dinucleotide (NAD(P)H), an endogenous fluorophore, was chosen as target^40^. NAD(P)H is mainly present in the mitochondria and directly involved in the ATP synthesis^41^ which are both damaged in the cells after DCF exposure^42,43^. Induced apoptotic and necrotic events were observed and then confirmed with flow cytometry analysis^44^. Besides, we investigated how the use of dimethyl sulfoxide (DMSO) as a co-solvent system in the electrospinning affects the scaffold morphology and its mechanical and drug eluting properties.

The demand for versatile, reliable *in vitro* models for drug screening and toxicity studies will increase in the years ahead^45^. One of the biggest limits of many *in vitro* models already available is that they can be highly specific and sensitive for particular applications, but they cannot be extended to other fields^46^. Thus, the challenge of creating innovative drug testing systems that can be analysed unmodified with non-invasive methodologies would provide models with increased precision and speed. In the present study we aimed to generate a model scaffold with electrospinning that allows a controlled and tuneable diffusion of encapsulated bio-active molecules and test them using a marker-independent, non-invasive approach.

## Results

### Generation of an electrospun scaffold enabling controlled and sustained Diclofenac release

**Figure 1:**
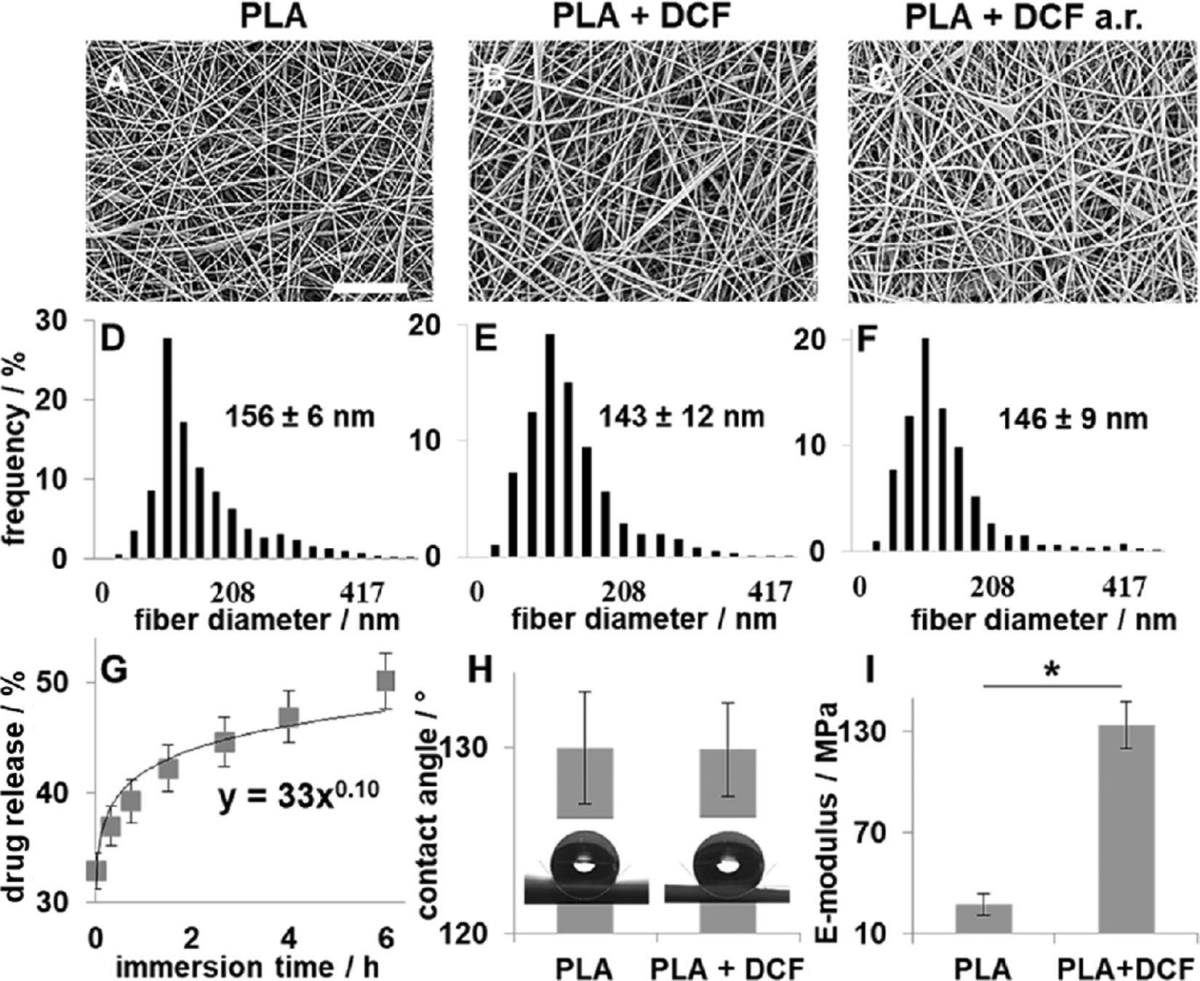
DCF-loaded scaffolds characterization. (**A**–**C**) SEM images of electrospun PLA (**A**), PLA + DCF (**B**) and PLA + DCF after release (a.r.). Scale bar = 50 µm. (**D**–**F**) Fiber size distribution of PLA (**D**), PLA + DCF (**E**), PLA + DCF a.r. (**F**). (**G**) Drug-release profile from PLA + DCF scaffolds. (**H**) Contact angle of PLA and PLA + DCF scaffolds. (**I**) E-modulus of PLA and PLA + DCF scaffolds.

DCF (11.8 wt %) was successfully encapsulated in a PLA scaffold via electrospinning. Scaffolds morphology and fibre sizes, before and after release (a.r.) were investigated using scanning electron microscopy (SEM) (Fig. 1A–C). The generated scaffolds had a uniform and random nanofibre orientation (Fig. 1A–F) and the mean diameter was not significantly affected by drug encapsulation (PLA: 156 ± 6 nm vs. PLA + DCF: 143 ± 12 nm, p = 0.39) nor by the drug-release (PLA + DCF a.r.: 146 ± 8 nm; vs. PLA, p = 0.36; vs. PLA + DCF, p = 0.61). The scaffold ability to release the drug was investigated by UV detection. The absorbance at 272 nm of the solution, in which the drug-loaded scaffold was immerged, was constantly monitored for 6 hours and measured again after 24, 48 and 72 hours. After 6 hours, 44 ± 5% of the encapsulated drug was released to a final DCFONa concentration of 0.71 ± 0.04 mg/mL in the extracts. Absorbance was constant after 24 hours and close to 100% (DCF released: 93 ± 4% leading to a DCFONa concentration of 1.5 ± 0.1 mg/mL in the extracts). For complete drug release a longer time as well as polymer degradation or erosion is required^47^. The obtained points were plotted against time and fitted using the equation developed by Peppas^48,49^ (Fig. 1G). The obtained release exponent (0.10 ± 0.02, Fig. 1G) suggests that the release undergoes a controlled diffusion^49^. The physical properties of the scaffolds were investigated as well since they may have an influence on the drug diffusion properties^50^. While scaffold hydrophobicity was not affected (contact angle PLA + DCF: 129 ± 3° vs. PLA: 131 ± 7°; Fig. 1H), the elastic modulus significantly increased when having DCF included (E-modulus; PLA + DCF: 133.8 ± 13.6 MPa vs. PLA: 27.4 ± 9.7 MPa, p = 0.021, Fig. 1I).

### FLIM-based non-invasive *in vitro* analysis of Diclofenac cytotoxicity towards human dermal fibroblasts

DCF-loaded electrospun PLA scaffold was used as a DCF delivery system to induce cell death in HDFs. Cells exposed to released DCF were imaged using multiphoton microscopy (MPM) coupled with fluorescence lifetime imaging microscopy (FLIM)^40^. Reduced (phosphorylated) nicotinamide adenine dinucleotide (NAD(P)H) was chosen as target for both analyses. NAD(P)H is mainly present in the mitochondria, directly involved in ATP synthesis^41^ and is damaged in the cells after DCF exposition^42,51,52^.

**Figure 2:**
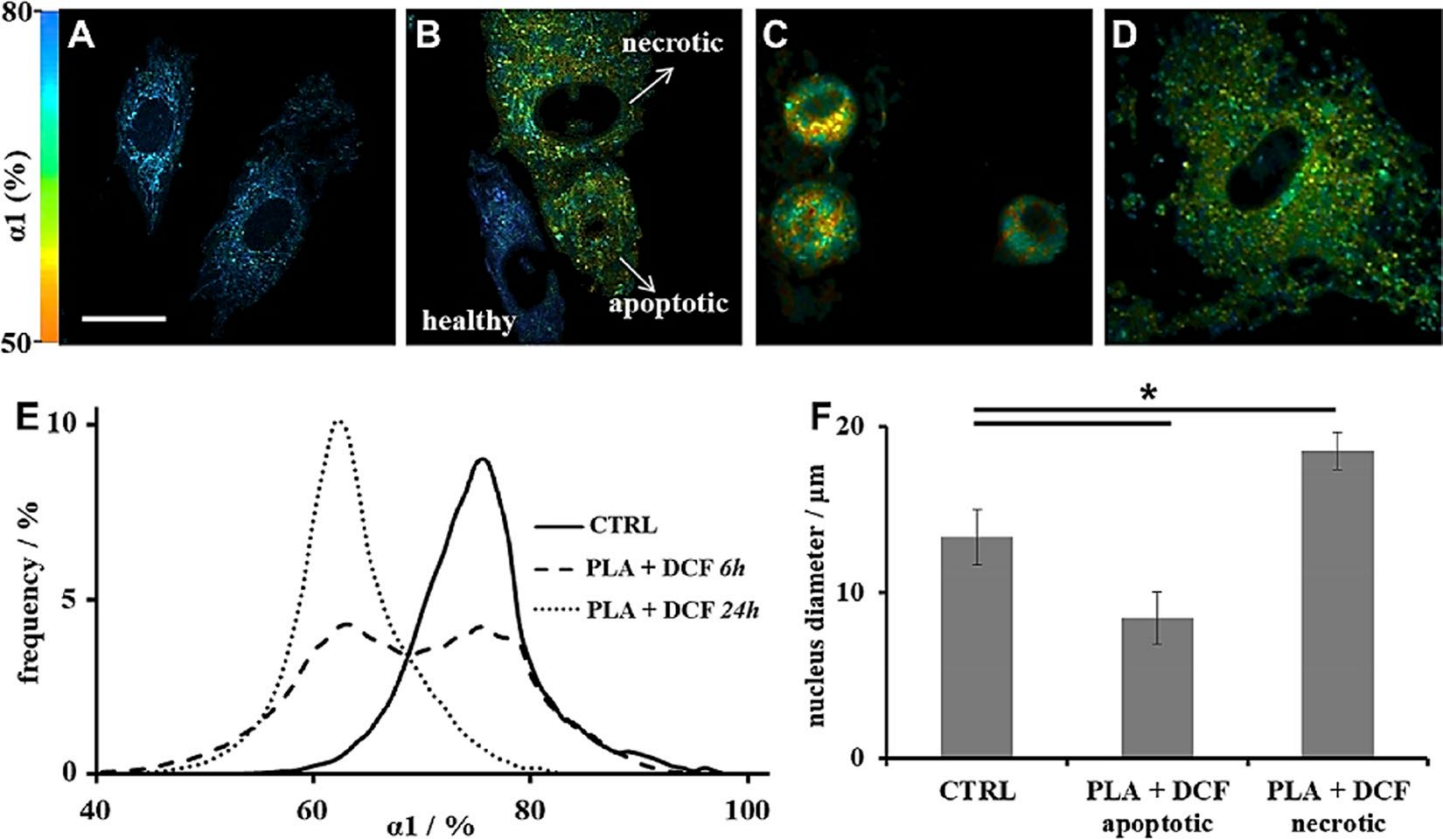
MPM and FLIM analysis of DCF-exposed HDFs. (**A**–**D**) FLIM colour-coded imaging of: untreated HDFs (**A**); DCF-treated HDFs after 6 h incubation time (**B**); DCF-treated HDFs assessed as apoptotic (**C**) and necrotic (**D**) after 24 h incubation time. Scale bar = 20 µm. (**E**) Histograms of α_1_% distribution after FLIM analysis. (**F**) Average nucleus diameter.

For FLIM analysis, a two exponential decay (Equation S1) was employed due to two different fluorescence lifetimes for free (τ_1_) and protein-bound (τ_2_) NAD(P)H^53,54^. α_1_% represents free NAD(P)H relative contribution to final lifetime values. 6 and 24 hours were chosen as incubation times. These two time points were chosen since within the first 6 hours we could apply Peppas equation (Fig. 1G), while after 24 hours no more DCF diffused from the scaffold. Via false colour-coded imaging based on the α_1_% values we were able to distinguish the DCF-affected cells from the untreated ones (CTRL, Fig. 2A–D). While the untreated cells were displayed in blue due to higher α_1_%s (Fig. 2A,B), the DCF affected ones were instead coloured red (Fig. 2B–D). Changes in free NAD(P)H contribution to the final lifetime suggest that the glycolytic activity of the DCF-damaged cells is strongly affected^40,52^.

In addition, cell morphology of the DCF-treated cells appears also altered when compared to the CTRL (Fig. 2A–D). Whereas after 6 hours treatment still a few non-effected and therefore elongated fibroblasts were imaged (Fig. 2B), the morphology of the DCF-treated cells after 24 hours treatment significantly changed towards a more rounded appearance (Fig. 2C,D). The α_1_% distributions additionally reflect these observations (Fig. 2E). Particularly, after 6 hours DCF-exposure two different cell populations could be detected, demonstrating that not all HDFs have been irreversibly damaged by DCF at this time point (Fig. 2E, dashed line). After 24 hours (Fig. 2E, dotted line), when compared to the CTRL (Fig. 2E, solid line), the cells show instead significantly lower α_1_% values (CTRL: 73.3 ± 4.9% vs. PLA + DCF 24 h: 62.1 ± 5.2%, p = 0.024).

Furthermore, a shift towards longer τ_1_ values could be observed (Fig. S1A). However, the changes were not significant compared to the untreated HDFs (CTRL: 0.63 ± 0.09 ns vs. PLA + DCF 6 h: 0.72 ± 0.11 ns, p = 0.3; CTRL: 0.63 ± 0.09 ns vs. PLA + DCF 24 h: 0.74 ± 0.12 ns, p = 0.35). τ_2_ values were also not affected by DCF (Fig. S1B). As previously described by Seidenari^55^, nuclear and cytoplasmic condensation is related to apoptosis (Fig. 2C), whilst swelling and vacuolation of the cytoplasm to necrosis (Fig. 2D). Apoptotic cells show a smaller nucleus than normal fibroblasts (PLA + DCF apoptotic: 8.5 ± 1.6 µm vs. CTRL: 13.3 ± 1.7 µm, p = 4e^−7^; Fig. 2F) while necrotic cells are larger^55–58^ (PLA + DCF necrotic: 18.6 ± 1.1 µm vs. CTRL: 13.3 ± 1.7, p = 1e^−7^, Fig. 2F).

**Figure 3:**
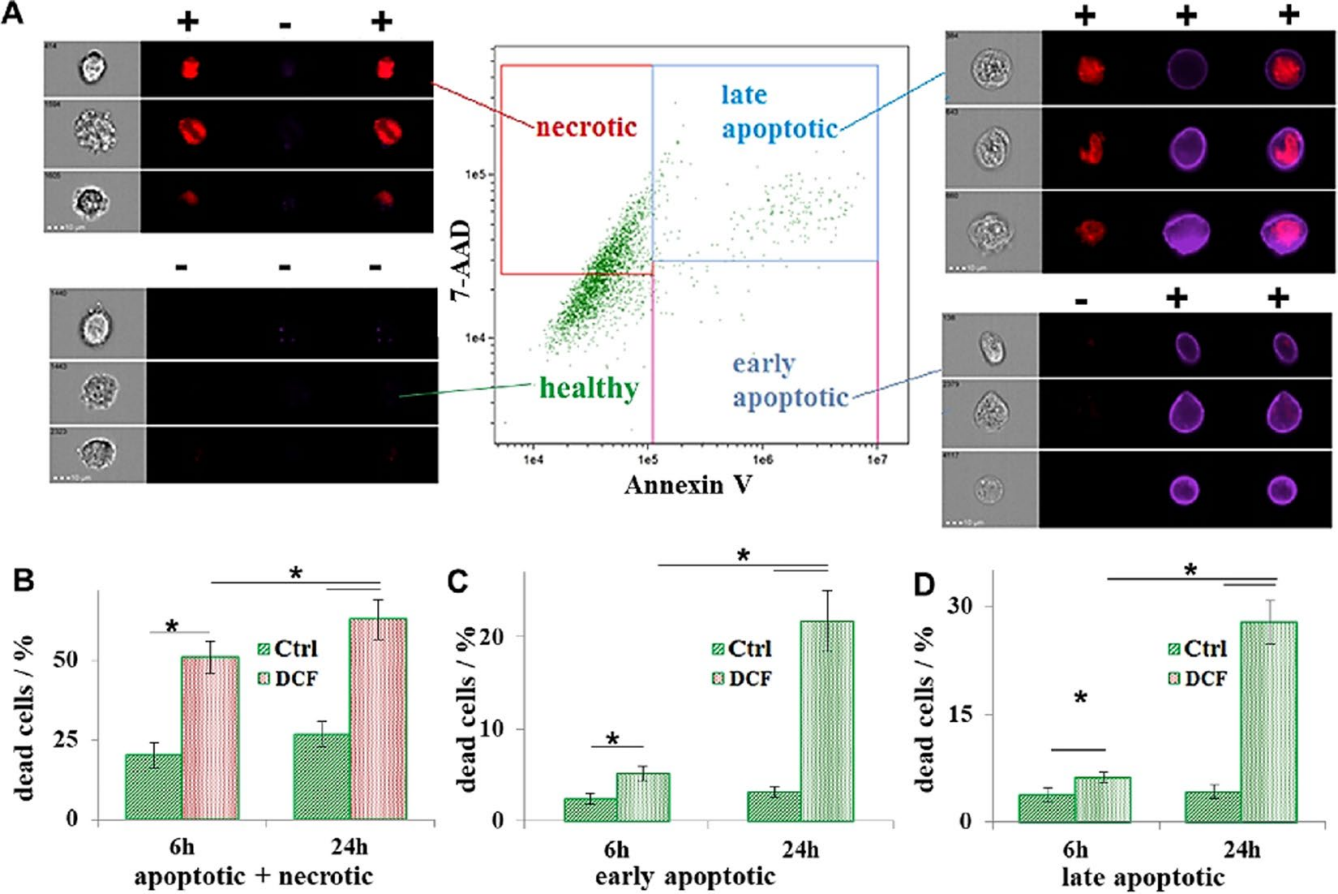
Imaging flow cytometry analysis. (**A**) Staining with AnnexinV (blue) and 7-AAD (red). (**B**–**D**) Data analysis of the cells after staining and counting (CTRL: green; DCF: red): total percentage of: dead cells (**B**); early (**C**) and late (**D**) apoptotic cells.

Flow cytometry was employed to confirm DCF-induced cell death (Fig. 3). AnnexinV was used as marker for apoptosis and 7-Aminoactinomycin D (7-AAD) for necrosis (Fig. 3A). Here, it was possible to distinguish between early (AnnexinV positive) and late apoptotic (AnnexinV and 7-AAD positive) cells (Fig. 3A). Imaging flow cytometry analysis showed that approximately 40% (39.4 ± 4.7%) of the cells undergo necrosis while approximately (Fig. 3B) 10% are either early (4.6 ± 0.7%, Fig. 3C) or late apoptotic (6.8 ± 0.4%, Fig. 3D) after 6 hours DCF exposure. After 24 hours, approximately 50% of the cells were either early (21.1 ± 3.8%, Fig. 3C) or late apoptotic (28.2 ± 2.3%, Fig. 3D). The total dead cells percentage was significant higher in the DCF-treated samples after 6 (DCF: 50.8 ± 5.3% vs. CTRL: 20.8 ± 2.2%, p = 0.008, Fig. 3B) and 24 hours (DCF: 56.7 ± 5.6% vs. CTRL: 25.1 ± 2.3%, p = 0.007, Fig. 3B), when compared to the control.

### Tuning the Diclofenac release from the electrospun PLA scaffold by adding DMSO

**Figure 4:**
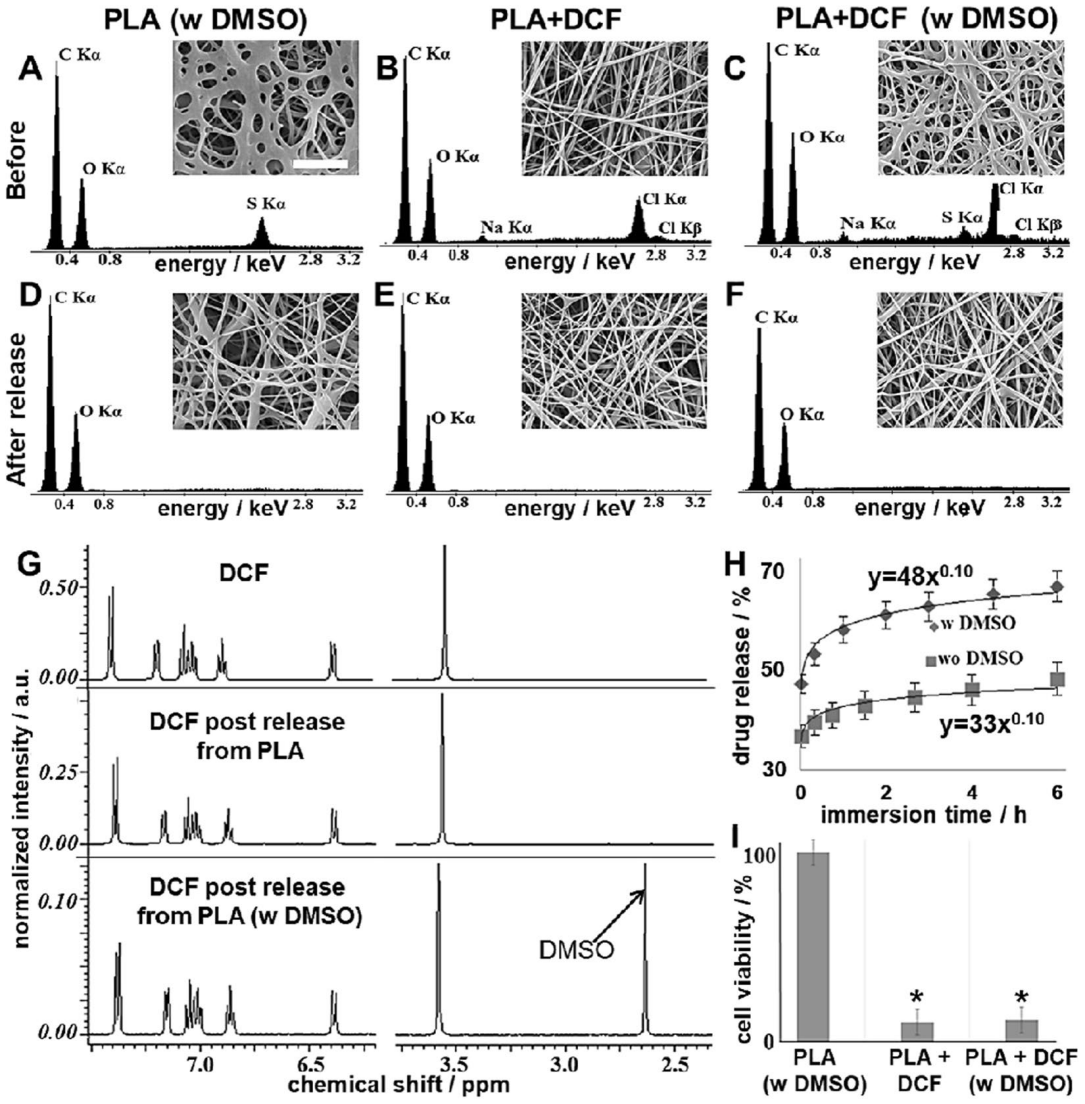
Characterization of DMSO-containing scaffolds. (**A**–**F**) SEM images (scale bar = 10 µm) and EDS spectra of the electrospun scaffolds before (**A**–**C**) and after (**D**–**F**) release. (**G**) 1H-NMR spectra in D2O of pure DCF (not encapsulated); extracts in PBS of DCF-loaded PLA and DCF-loaded PLA DMSO scaffolds after water removal by freeze-drying. (**H**) Drug-release profile and fitting according to Peppas-Korsmeyer model for PLA + DCF (squares) and PLA + DCF w DMSO (rhombi). (**I**) MTS assay for cytotoxicity assessment.

As we successfully demonstrated non-invasive monitoring of cell death, we were furthermore interested whether it is possible to control and tune the release of DCF from the electrospun scaffolds in order to provide a model with tuneable characteristics. We aimed to enhance DCF initial release from our system by adding DMSO. The overall scaffold morphology appeared to be altered, when adding DMSO to the electrospinning solution (Fig. 4A–C). The DMSO-containing fibres were spread or merged; however, no beads formed and the structure was still consistent over the entire scaffold. Interestingly, after the release experiments, DMSO-containing samples regained the fibrous structure typical for electrospun scaffolds without DMSO (Fig. 4D–F). Changes in the scaffold morphology were due to DMSO encapsulation and its subsequent release. To confirm our hypothesis, we demonstrated DMSO and DCF encapsulation with energy dispersive X-ray spectrometry (EDS) and their release with ^1^H-NMR and EDS as well. We used pure PLA as a polymer, polyester composed of Carbon (C), Hydrogen (H) and Oxygen (O). DMSO contains Sulphur (S), while DCF Sodium (Na) and Chlorine (Cl). Thus, we could correlate DMSO presence to S peak on the EDS spectrum, and Cl and Na signals with the effective DCF encapsulation (Fig. 4A–C). After the release experiments, only C and O signals could be detected, showing that DMSO and DCF were released from the scaffold (Fig. 4D–F; Fig. S2), with a final content lower than 10% weight^59^. Effective DCF release from the scaffolds was determined also using reverse phase (RP)-HPLC (Fig. S3) and ^1^H-NMR (Fig. 4G). In addition, we investigated the release mechanism using Peppas equation^48,49^. Interestingly, DMSO did not alter DCF diffusion mechanism but significantly enhanced its initial burst release (Fig. 4H). In particular, no significant difference (p = 0.82) in the release exponent could be found between the samples containing DCF alone, and the ones containing DCF and DMSO, with obtained values of 0.10 ± 0.02 and 0.10 ± 0.03, respectively (Fig. 4H). DMSO enhanced instead the DCF burst release, with obtained values for the release rate constants of 0.48 ± 0.03 h^−0.1^ and 0.33 ± 0.02 h^−0.1^ (p = 0.003), with the presence or absence of DMSO, respectively (Fig. 4H). Consequently, 63 ± 6% of the encapsulated drug diffused from the scaffold after 6 hours to a DCFONa concentration of 1.00 ± 0.06 mg/mL in the extracts. In order to exclude any additional cytotoxic effects of DMSO, we assessed *in vitro* cytotoxicity by performing a MTS (3-(4,5-dimethylthiazol-2-yl)−5-(3-carboxymethoxyphenyl)-2-(4-sulfophenyl)-2H-tetrazolium) assay with scaffold extracts. Despite of the altered fiber morphology (Fig. 4A–C), the DMSO in the electrospun scaffold did not impact cell viability (Fig. 4I). In contrast, and as expected, DCF caused cell death independent of DMSO presence. Cell viability was also analyzed after treatment with free DCFONa (Fig. S4). As expected, cell viability was around 0% comparable to the SDS control.

## Discussion

Improved *in vitro* models are required to help to identify and investigate candidate molecules for pharmaceutical development^60^. *In vitro* studies are generally less expensive, faster and more flexible than regulated *in vivo* tests^61^. Nevertheless, the limited reliability and *in vivo* reproducibility of existing *in vitro* tests place great emphasis on the need for more realistic models. Over the last decades, many investigators have been researching on *in vitro* assays that should enable informed strategic decisions already during screening processes, thus avoiding entire animal testing^60,61^. One of the biggest limits of the nowadays available *in vitro* systems by assessing the toxicity of drugs is the necessity to work with specific concentrations of the investigated substance. This approach is good to assess dangers and risks related to the hazards, but it doesn’t really reproduce the therapeutic situation^60^. Controlled delivery over time can in this way better mimic the drug bioavailability after its administration, and can also help to reproduce the real daily dosages. Nano-delivery systems represent a great future perspective in this field, and are gaining a constantly growing interest among scientists^62,63^.

In this work, we could effectively encapsulate DCF in a PLA electrospun scaffold, finally having a system that allowed us to obtain a controlled drug delivery over 24 hours. Once proved the effective controlled DCF release, using MPM coupled with FLIM we could image changes in morphology and metabolism of HDFs exposed to the scaffold extracts. FLIM analysis showed changes in the metabolism of HDFs after their exposure to the cytotoxic DCF, in terms of a significant decrease in the contribution of free NAD(P)H (α_1_%) to the glycolytic activity^53,54^. Profiting from the different α_1_%s, DCF-affected cells could be distinguished from the untreated ones by false-colour coding. After DCF exposure, cells also showed a different morphology compared to untreated HDFs. Morphological changes were attributed to induced apoptosis and necrosis. The occurring of these events after the DCF release from the scaffolds was confirmed with flow cytometry^44^. Necrotic cells were prevalent after 6 hours treatment, while apoptotic after 24 hours. This is in accordance with previously reported works showing that necrotic events occur earlier then apoptotic events after cell exposure to toxins^56–58^. According to these data, we could show that both apoptosis and necrosis are induced in HDFs, when exposed to DCF. A well-recognized effect of high concentrations of DCF is rapid and concentration-dependent cellular energy depletion^28^. In addition to its action as a non-selective COX-inhibitor, DCF inhibits the outward cellular transport of lactate, thus reducing the glucose uptake. As a result, both mitochondrial and glycolytic ATP production is inhibited^43,64^. Masubuchi *et al*.^28^ have demonstrated that a relevant ATP depletion occurs in rat hepatocytes, when exposed to high DCF concentration (up to 100 µM). The ATP depletion is almost complete when working with DCF 500 µM^29^. Drug-induced oxidative stress can primarily recruit rescue pathways, but, if sustained, the stress can cause mitochondrial injury^65,66^. Thus, the cells are usually not able anymore to repair the damage, and apoptotic or necrotic events mainly occur^67–69^.

Furthermore, in this study, we demonstrated a possibility to enhance the initial release of DCF from our system by simply adding DMSO without changing the DCF content in the scaffold nor the drug-release profile. DMSO is a polar high boiling solvent and has been widely used as a co-solvent system for electrospinning to improve the solubility of molecules and polymers, which cannot be easily dissolved in common organic solvents. Thus, by adding DMSO electrospinnability of the final solutions can be dramatically enhanced^70–72^. Besides, DMSO is efficiently employed as a skin penetration enhancer in many pharmaceutical formulations^73,74^. For these reasons it represents an interesting candidate in the production of electrospun scaffolds as drug delivering systems^71,72^. We could observe in our study that the addition of DMSO doesn’t alter the drug diffusion mechanism but only enhances the initial burst release of DCF. Nevertheless, the fibrous morphology of the scaffold appears clearly altered according to SEM observations, when having DMSO in the electrospun materials. The DMSO containing scaffolds seem crosslinked or merged^75^. In this case, altered fiber and pore sizes did not affect cell behaviour as described in previous studies^76,77^, since the cells are not in direct contact with the scaffold.

It has been already shown that by changing the amount of the drug loaded in the electrospun fibers, the mechanism of the release and the final drug solubility in the extracts are also affected^78,79^. Interestingly, despite altering the fiber morphology we could show that, without changing the drug loading, the presence of DMSO helps to enhance the initial burst release of the drug without altering the subsequent diffusion mechanism according to the model proposed by Peppas *et al*.^48,49^. Furthermore, also the amount of DCFONa in the scaffold extracts after 24 hours did not change when having DMSO as well. Moreover, after the release experiments, the DMSO containing samples regained the fibrous structure, which is typical for electrospun scaffolds. The changes in scaffold morphology were related to the encapsulation of DMSO and its subsequent release according to our observations.

To confirm our hypothesis we proved DMSO and DCF encapsulation with EDS, and their release with ^1^H-NMR and EDS as well. EDS analysis has already been employed to investigate the elemental composition of electrospun scaffolds^80^. In our case, we used pure PLA, a polyester composed of only C, H and O. On the other side, DMSO contains S as well, while DCF Na and Cl. Thus, we were able to correlate the presence of DMSO with the presence of S peak on the EDS spectrum, and Cl and Na signals to the effective DCF encapsulation. Interestingly, after the release experiment (24 hours PBS immersion) only C and O signals could be detected from all the scaffolds, suggesting the diffusion of both DMSO and DCF from the starting functionalized materials, with a final content lower than 10% in weight, due to EDS detection limits^59^. With ^1^H-NMR we could also demonstrate that DCF and DMSO were released unmodified from the electrospun scaffolds. This analysis was necessary in order to demonstrate that the encapsulated molecules effectively undergo a diffusion and not degradation or modification during the whole processing.

After characterizing the release properties of the modified scaffolds, we could demonstrate that cytotoxic effects were related to the diffusion of the unmodified DCF according to the results of an accredited MTS assay. Instead, the presence of DMSO in the scaffolds did not affect PLA biocompatibility, probably because of its low amount^81–83^. This result is interesting and suggests that DMSO could be effectively encapsulated in electrospun scaffolds to tune their properties, and enhance drug diffusion from the delivery systems. According to our idea, the drug-loaded scaffolds can be used as models to reproduce therapeutic doses, besides ensuring controlled drug diffusion over time.

To conclude, we encapsulated DCF in a PLA scaffold via electrospinning and produced a system for a controlled drug release. We used the drug-loaded scaffold as a delivery system to investigate *in vitro* DCF toxicity on HDFs. We demonstrated that DCF induces both apoptosis and necrosis in HDFs. Changes in cell morphology and metabolism were non-invasively detected using MPM coupled with FLIM. A big limit of many already existing *in vitro* systems is that the cells get damaged or die directly after the analysis, and so cannot be monitored over time^84^. Using MPM and FLIM, we were instead able to image and analyse the cells at different time points. Moreover, we demonstrated that DMSO is an attractive additive to enhance initial burst release of drugs from electrospun scaffolds without affecting their biocompatibility. In this way, we believe that the here described model could represent an interesting way to control the diffusion of encapsulated bio-active molecules and test them using a marker-independent, non-invasive approach. In the next future we aim to test the here presented model with other drugs and more adequate *in vitro* cell systems^85,86^. Especially 3D models^86–88^ would better mimic the *in vivo* behaviour than monolayer cell cultures.

## Materials and Methods

### Scaffolds production and characterization

PLA (M_n_ 59,000, M_w_ 101,000) and DCF were purchased by Sigma-Aldrich (Steinheim, Germany). 1,1,1,3,3,3-Hexafluoro-2-propanol (HFP, Iris Biotech, Marktredwitz, Germany) was used as the solvent. When using DMSO (≥99.9%, for molecular biology, Sigma-Aldrich) 19:1 was the final HFP:DMSO volume:volume ratio. A 15% PLA (+2% DCF) w/v concentration was used (final volume: 1.2 mL). Electrospinning was performed with a customized device. Flow rate was set to 4 mL/h and voltage to 20 kV, using an 18 G needle at a collector distance of 18 cm. For drug release experiments punches (Ø = 28 mm, wt = 27 mg) of drug-loaded scaffolds were immerged in 2 mL of 1X Phosphate buffer saline (PBS, Gibco™ by Life Technologies GmbH, Darmstadt, Germany) solution. The initial DCF content in each punch was estimated considering a 15:2 PLA:DCF wt:wt ratio and set to 100% when expressing the release in percentage^48,49^. The scaffold-containing solution was then incubated by 37 °C and, at different time points, 200 µL of the supernatant was taken to read the absorbance (272 nm). Scaffolds morphology was determined using a scanning electron microscope (1530 VP, Zeiss, Jena, Germany), after platinum sputter coating. Images were acquired at a working distance of 8 mm and a voltage of 15 kV. The ImageJ® software supplied with the DiameterJ plug-in was used for fibre diameter analysis. EDS analysis of uncoated samples were performed on a scanning electron microscope (FEI E-SEM XL30) equipped with energy dispersive X-ray spectrometer (EDAX GEMINI 4000). Voltage was set to 30 kV and working distance to 10 mm. Contact angle and Young’s modulus were determined as previously described^40^. RP-HPLC analyses were performed on a Shimadzu (Kyoto, Japan) automated system supplied with a Jupiter C5 column (Phenomenex, 250 ×4.6 mm, 5 µ, 300 Å) and UV detection. A binary gradient between double distilled water, with 0.1% wt trifluoroacetic acid (Romil Ltd, Cambridge, UK), and HPLC purity grade acetonitrile (Romil) was used. Products were eluted with a gradient of acetonitrile from 5–70% over 45 min. Flow rate was set to 1 mL/min. Reported chromatograms were recorded at 272 nm. ^1^H-NMR spectra were acquired on an Inova 500 NMR spectrometer (Varian, Agilent technologies, Palo Alto, USA), equipped with a 5 mm triple resonance probe and z-axial gradients operating at 500 MHz for ^1^H nuclei. Solvent was deuterium oxide (D_2_O, Sigma-Aldrich) with 4,4-dimethyl-4-silapentane-1-sulfonic acid (Sigma-Aldrich) as standard (chemical shift (δ): 0.00). Spectra were processed with ACD®/NMR Processor (ACD, Toronto, Canada).

### Cell culture and seeding

All research was carried out in compliance with the rules for investigation of human subjects, as defined in the Declaration of Helsinki. We obtained informed consent from the patient. This study was carried out in accordance with the institutional guidelines and was approved by the local research Ethics Committee (the ethics commission of the Landesärztekammer F-2012-078). Human dermal fibroblasts (HDFs) were isolated by enzymatic digestion as previously described^80^. Cells were cultured in Dulbecco’s modified eagle medium (DMEM, with L-Glutamine, Gibco™, Life Technologies GmbH, Darmstadt, Germany) supplemented with 10% fetal calf serum (FCS, PAA Laboratories, Pasching, Austria) and 1% penicillin/streptomycin (100 U/mL Penicilium and 100 μg/mL Streptomycin, Life Technologies GmbH). Cells were cultured in an incubator at 37 °C and in a 5% CO_2_ atmosphere. Cell culture medium was changed every 3 days and cells were passaged or seeded using trypsin-EDTA (15090046, PAA Laboratories) at approximately 70% confluence.

### MPM and FLIM imaging

Images were acquired with a custom built 5D multiphoton FLIM microscope (JenLab GmbH, Jena, Germany). Two-photon excitation was generated using a Ti: Sapphire femtosecond laser (MaiTai XF1 Spectra Physics, United States, Santa Clara). Fluorescence lifetime signals of NAD(P)H were recorded using time correlated single photon counting at an excitation wavelength of 710 nm at a laser power of 18 mW. The spectral emission filter for NAD(P)H ranged from 425 to 509 nm. FLIM data were recorded at an acquisition time of 180 seconds for 512 × 512 pixels (690 μs/pixel) with 64 time channels. The instrument response function was recorded using urea crystals (Sigma-Aldrich) at an excitation wavelength of 920 nm and a laser power of 4.5 mW for 120 s. HDFs were imaged on glass bottom dishes (Ibidi®, 35 mm) with a density of 5 ×10^4^ cells per dish. After 24 hours, the medium was removed and 2 mL of fresh DMEM (+10% FCS) was added. For drug release experiments, drug-loaded scaffolds and non-loaded control scaffold punches (Ø = 28 mm, wt = 27 mg; previously sterilized for 2 h with UV light) were placed in the medium. The FLIM images were analysed using the SPCImage software (Becker & Hickl GmbH, Berlin, Germany). A biexponential decay fitting model (Equation S1) was employed at each pixel since NAD(P)H has two different lifetimes represented by τ_1_ and τ_2_^53,54^. A χ^2^ < 1.1 was accepted for fitting. Nucleus diameter was evaluated using the ImageJ® software.

### *In vitro* cytotoxicity

For imaging flow cytometry analysis, AnnexinV (AnnexinV Apoptosis Detection Kit eFluor™ 450) and 7-AAD (7-AAD Viability Staining Solution) were purchased from eBioscience™ (Thermo Fischer Scientific, Darmstadt, Germany). For each group, cells were obtained from three different standard cell culture dishes previously analysed with FLIM. Cells were first recovered and then stained with AnnexinV and 7-AAD as per manufacture protocol, before analysis on an ImageStreamX Mark II (Amnis, Seattle, USA) with INSPIRE instrument controller software. Resultant data, obtained from at least 10000 cells per sample, were analysed with the IDEAS software (Amnis). Samples were gated on single cells in focus (40X magnification) and analysed for AnnexinV and 7-AAD.

The MTS ([3-(4,5-dimethylthiazol-2-yl)-5-(3-carboxymethoxyphenyl)-2-(4-sulfophenyl)-2H-tetrazolium]) assay was performed according to an ISO 10993-5 accredited protocol. HDFs were exposed to an extract of the samples. Briefly, the electrospun scaffolds were sterilized with UV (254 nm) for 2 hours. 6 cm^2^ of each sample was then incubated in 1 mL FCS- and antibiotic-free DMEM medium for 24 hours. Each extract was prepared in triplicate. HDFs seeded in 96-well plates (2000 cells/well) and were then exposed for 24 hours to the extracts (released drug from the scaffolds) supplied with 10% FCS. The extraction medium was removed, the cells washed twice with PBS 1X (Gibco™ by Life Technologies GmbH) and a MTS assay (CellTiter 96Aqueous One Solution Cell Proliferation Assay, Promega, Mannheim, Germany) was performed as per the manufacturer’s protocol. Briefly, 20 µL of MTS solution was added to 100 µL of the scaffolds extracts. After 35 minutes incubation at 37 °C, the absorbance of each well was measured at 492 nm using a TECAN® Infinite 200 Reader. The test was performed for a blind, a negative control (untreated HDFs exposed to fresh DMEM supplement with 10% v FCS) and sodium dodecyl sulphate (SDS, Life Technologies GmbH, 1% w/v in DMEM) or pure DCFONa (1.5 mg/mL in DMEM supplemented with 10% v FCS) treated positive controls. For analysis, the negative control was set to 100%.

### Data analysis

Reported graphs were plotted using Microsoft™ Excel. All data are presented as mean ± standard deviation (n = 4 unless stated otherwise). Student’s t-test (two-tailed, unpaired) was performed where appropriate to determine statistically significant differences between two groups. In experiments where measurements were performed before and after the release of DCF from the scaffolds, Student’s t-test (two-tailed, paired) was applied. Statistical significance was set at p < 0.05.

## Supplementary information

Dataset 1
