# Supplementary material for: Controlled and tuneable drug release from electrospun fibers and a non-invasive approach for cytotoxicity testing
Source: Sci Rep. 2019 Mar 5;9:3446. doi: 10.1038/s41598-019-40079-7 (PMC6401126; doi:10.1038/s41598-019-40079-7)
Supplement: Supplementary file 1 — Dataset 1 [file 41598_2019_40079_MOESM1_ESM.pdf]

Supplementary material

Controlled and tuneable drug release from electrospun fibers and a non-invasive approach for cytotoxicity testing

G. Piccirillo[a,b], D. A. Carvajal Berrio[b], A. Laurita[a], A. Pepe[a], B. Bochicchio[a], K. Schenke-Layland[b,c,d], S. Hinderer[b,c*]

[a]Dept. of Science, University of Basilicata, 85100 Potenza, Italy; [b]Dept. of Women's Health, Research Institute for Women's Health, Eberhard-Karls-University Tübingen, 72076 Tübingen, Germany; [c]Dept. of Biophysical Chemistry, Natural and Medical Sciences Institute (NMI) at the University of Tübingen, 72770 Reutlingen, Germany; [d]Dept. of Medicine/Cardiology, Cardiovascular Research Laboratories, David Geffen School of Medicine at UCLA, Los Angeles, CA, USA.

* Corresponding author: Svenja Hinderer, NMI Reutlingen, Department of Biophysical Chemistry, Markwiesenstr. 55, 72770 Reutlingen, Germany; Fax: +49-7121-51530-16; Tel: +49-7121-51530-802; E-mail: svenja.hinderer@nmi.de

$$I(t) = \alpha_1 e^{-\tau_1} + \alpha_2 e^{-\tau_2} + C$$

Equation S1. Two exponential decay fitting used for the FLIM analysis. α_1 represents the free NAD(P)H lifetime time while α_2 the protein bound NAD(P)H one.

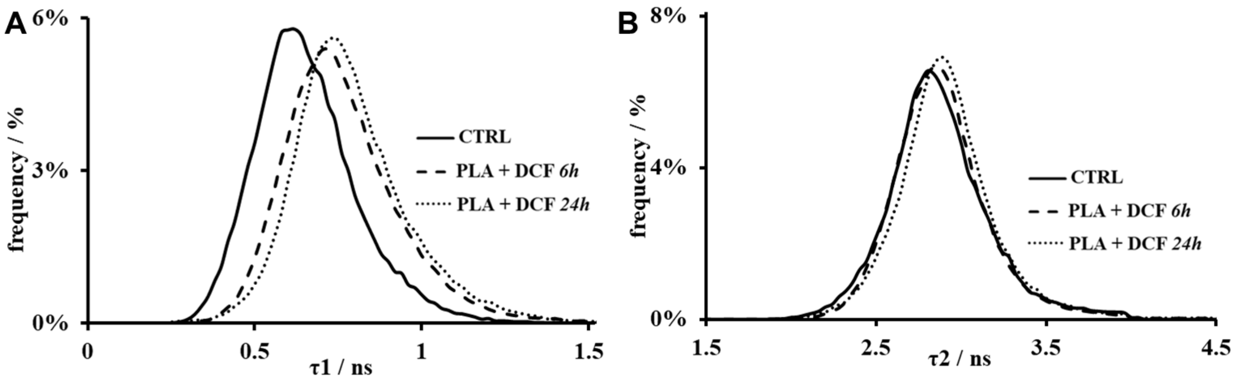

Fig. S1. Histograms for the distribution of τ_1 (A) and τ_2 (B) values

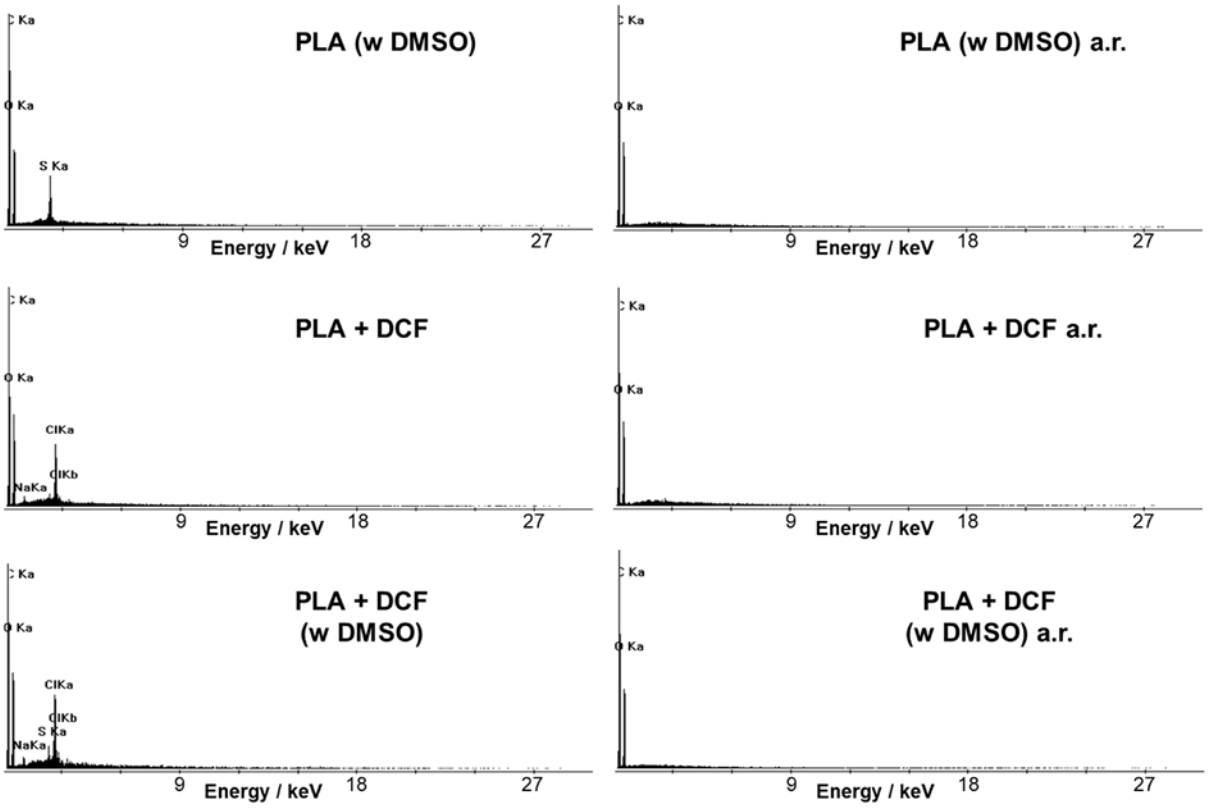

Fig. S2. Full EDS spectra of the electrospun scaffolds (a.r.=after release).

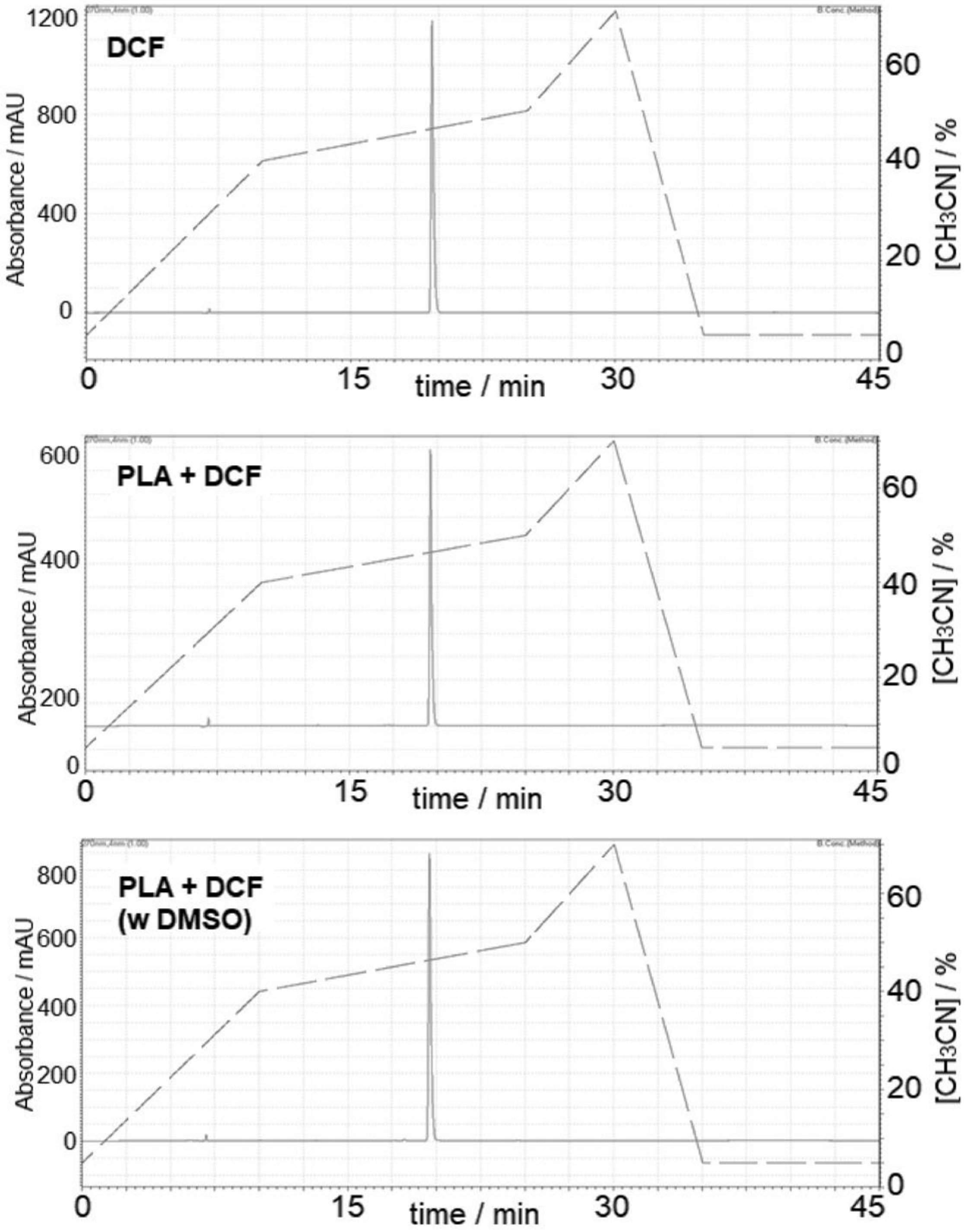

Fig. S3. RP-HPLC chromatograms. From above: DCF 1 mg/mL in PBS; DCF in PBS after its release from a PLA scaffold; DCF in PBS after its release from a DMSO-containing PLA scaffold. Dashed line represent acetonitrile gradient.

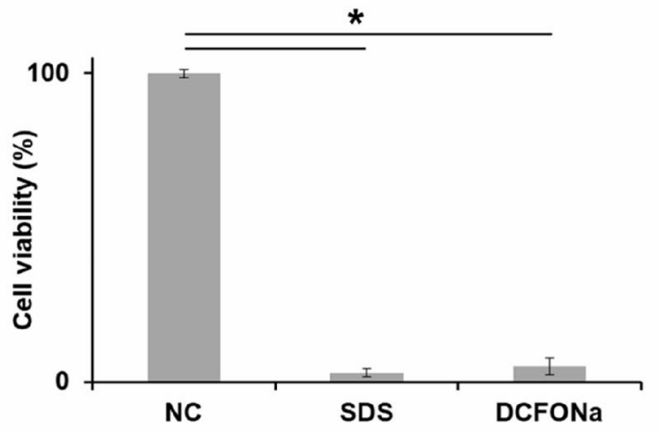

Fig. S4. MTS-assay. Cell viability after treatment with free DCFONa. *$p<0.05$
